# An Integrated ISFET Sensor Array

**DOI:** 10.3390/s91108831

**Published:** 2009-11-04

**Authors:** Kazuo Nakazato

**Affiliations:** Department of Electrical Engineering and Computer Science, Graduate School of Engineering, Nagoya University, Furo-cho, Chikusa-ku, Nagoya, Aichi 464-8603, Japan; E-Mail: nakazato@nuee.nagoya-u.ac.jp; Tel.: +81-52-789-3307; Fax: +81-52-789-3139

**Keywords:** ISFET, biosensor array, source-drain follower, CMOS biotechnology

## Abstract

A monolithically integrated ISFET sensor array and interface circuit are described. A new high-density, low-power source-drain follower was developed for the sensor array. ISFETs were formed by depositing Au/Ti extended-gate electrodes on standard MOSFETs, then thin silicon nitride layers using catalytic chemical vapor deposition and/or SU-8 protective layers were formed on the extended-gate electrodes. Applications for the array include: (1) pH detection by statistical distribution observing time and space fluctuations; (2) DNA detection using thiol-modified or silane-coupled oligonucleotides; (3) bio-image sensing by converting photons to electrons using Photosystem I of *Thermosynechococcus elongatus*, and sensing the converted electric charges by ISFETs.

## 1. Introduction

In view of the growing concerns about issues such as food security, healthcare, evidence-based care, infectious disease, and tailor-made medicine, the development of a portable gene-based point-of-care testing (POCT) system has become an important task. Conventional DNA chips are based on optical detection, whereby the target DNA is labeled with fluorophors and detected by optical laser scanner. This method is, however, problematic in that it requires a technically trained operator and expensive, nonportable equipment.

For a portable gene-based POCT system that anyone can operate anywhere and obtain immediate results, a new biosensor chip must be developed. Electrical detection using CMOS integrated circuits has great potential since it eliminates the labeling process, achieves high accuracy and real-time detection, and offers the important advantages of low-cost and small-sized equipment. Several methods have been investigated based on the detection of change in capacitance [1-3], current [4-6], and electric potential [7-10] by molecular interaction. Among them, the detection of electric potential change based on an ion-sensitive field-effect transistor (ISFET) [11] has shown excellent sensitivity for ion concentration [12], penicillin [13], glucose [14], urease [15], neuronal activity [16], extracellular recording [17], specific DNA sequence including single nucleotide polymorphisms (SNPs) [18-21], and so on. The operating principle of the ISFET is simple. Specific molecules are selectively taken into a probe layer on the FET channel, which detects the molecular charge in the probe layer. In the case of DNA detection, the probe is single-stranded (ss) DNA with a known sequence, immobilized on the substrate. When the target ss-DNA is supplied, hybridization occurs if the target DNA is complementary to the probe DNA. With or without specific hybridization can be detected by the difference in charge since a nucleotide has a negative charge on the phosphate group.

**Figure 1.**
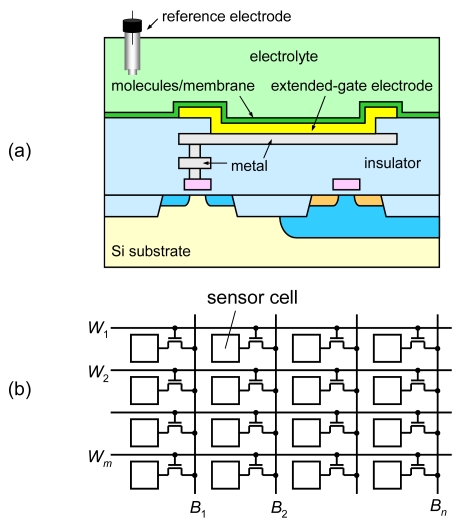
Integrated ISFET sensor array. (a) Schematic cross section of a sensor cell and (b) matrix array arrangement.

This type of ISFET has been studied as a discrete transistor controlled by external equipment [22]. Our target is a monolithically integrated sensor array, as shown in Figure 1, which detects all possible biomolecular interactions simultaneously. In each sensor cell, different kinds of probes can be formed for parallel detection. In addition, the same kind of probe can be used to observe the time development of spatial distribution of biomolecular interactions as well as to improve the detection accuracy since biomolecular interactions are a stochastic process.

Another application is in image sensors by incorporating biological materials with an ISFET sensor array [23]. The quantum yield of the photoelectric conversion of photosynthesis, which is a chemical process occurring in plants, algate, and cyanobacteria, is 100% [24]. In a recently developed process, a molecular wire is plugged directly into a biological photosynthetic system to efficiently conduct electrons to a gold electrode [25]. By incorporating such biological materials with an integrated circuit, a completely new type of device can be realized.

For the integrated sensor array, the structure must be compatible to complementary metal-oxide-semiconductor (CMOS) integrated circuits. Employment of extended-gate electrodes is one solution, as shown in Figure 1(a). Molecules and/or membrane are formed on the extended-gate electrodes. Our goal is realization of a million-sensor array on a single chip. One sensor must occupy a small area, and consume low power. Since the detection signal is in the order of 10 mV, high accuracy is required. To meet these targets, we propose a new integrated sensor circuit, CMOS source-drain follower [26,27].

## 2. CMOS Source-Drain Follower

**Figure 2.**
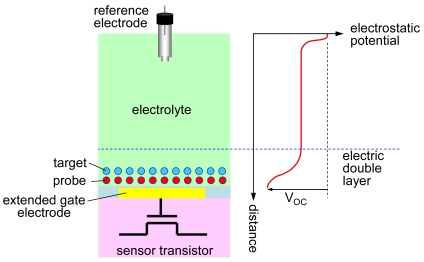
Detection of open-circuit voltage (Voc) at extended-gate electrode.

The detection scheme using an extended-gate electrode is shown in Figure 2. In one configuration, the electrode is in direct contact with the electrolyte and molecules. In another configuration, it is an electrically insulated ‘floating gate.’ Open-circuit voltage (Voc) at the extended-gate electrode relative to the reference electrode is detected at the MOSFET gate. When electrons are exchanged between the extended-gate electrode and electrolyte/molecules, Voc is determined by the redox potential (Nernst equation). When electrons are not exchanged, Voc is determined by the electric charge (Poisson equation) inside the electric double layer, the thickness of which is several nanometers depending on the electrolyte ion concentration. In the CMOS circuit, the detection signal is the extended-gate voltage, which is the gate voltage *V_IN_* of the sensor transistor. The detection of Voc requires high input impedance of the CMOS circuit, much higher than the impedance between the extended-gate and reference electrodes.

**Figure 3.**
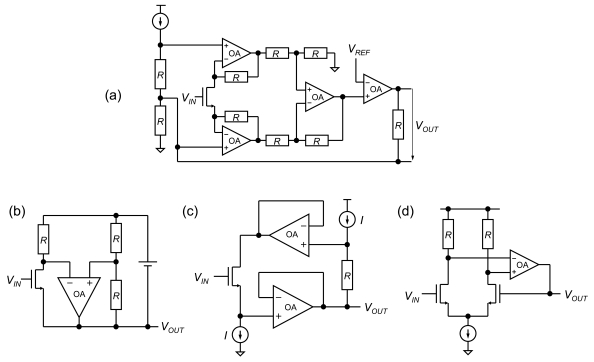
Conventional source-drain followers. (a) Instrumentation amplifier [7,22], (b) bridge type [8,22], (c) two op amps [9,19], (d) differential amplifier [22].

One of the circuits commonly used for ISFET sensors is a source-drain follower, where both the gate-source voltage and gate-drain voltage of the sensor transistor are maintained at constant values [22]. The source-drain follower has the merit of not influencing the sensing system since the input impedance is infinite for both DC and AC signals. Such source-drain followers have been constructed by op amps, as shown in Figure 3. However, these circuits are not suitable for an on-chip sensor array because of their large occupied area and high power consumption.

**Figure 4.**
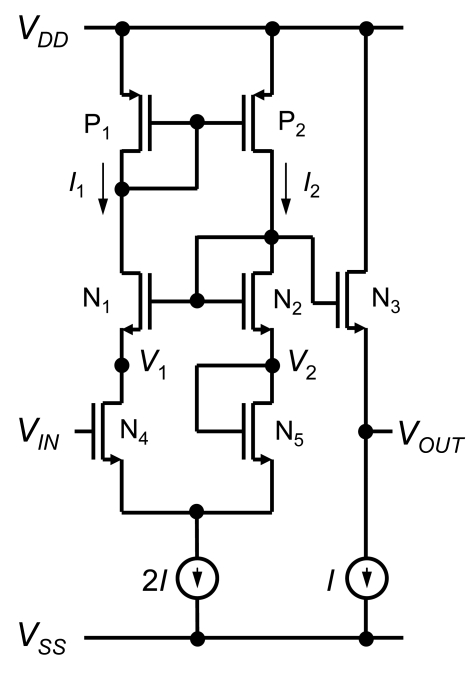
Basic CMOS source-drain follower.

We propose a CMOS source-drain follower, the basic circuitry of which is shown in Figure 4 [26,27]. The circuit consists of a PMOS current mirror {P_1_, P_2_}, NMOS source followers {N_1_, N_2_, N_3_}, pair of source-coupled transistors {N_4_, N_5_}, and current sources. The PMOS current mirror {P_1_, P_2_} keeps currents *I*_1_ and *I*_2_ equal to *I*. The NMOS source followers {N_1_, N_2_, N_3_} keep voltages *V*_1_ and *V*_2_ equal to *V_OUT_*. The sensor transistor, N_4_, detects the extended-gate electrode voltage *V_IN_*. In the pair of source-coupled transistors {N_4_, N_5_}, the drain current, drain voltage, source voltage, and body voltage are equal, so the gate voltage of N_4_ must be equal to the gate voltage of N_5_, which is *V*_2_. Thus, the output voltage is equal to the input voltage, and this circuit works as a voltage follower with high input impedance *Z_in_* and low output impedance *Z_out_* given by the inverse of the transconductance of N_3_. The power gain ∼ *Z_in_*/*Z_out_* is extremely high. Since the sensor area and power consumption are limited, the output impedance *Z_out_* is around 10–100 kΩ. A benefit of the voltage follower is that the output voltage is independent of device parameters such as threshold voltage and environmental conditions such as temperature. The mismatch of transistor characteristics in groups {P_1_, P_2_}, {N_1_, N_2_, N_3_}, and {N_4_, N_5_} can be reduced by means of a close, symmetrical layout. This circuit also works as a source-drain follower for sensor transistor N_4_. The gate-drain voltage is fixed at 0 V, and the gate-source voltage is fixed at *V_Tn_* + (2*I* (*L*/*W*)_N4_ /*μ_n_C_ox_*)^1/2^ when current *I* is kept constant, where *V_Tn_* is the threshold voltage of the n-channel MOSFET, *W* and *L* are channel width and length of transistor, respectively, *μ_n_* is the electron mobility, and *C_ox_* is the gate oxide capacitance per area. The source-drain follower has several advantages including the provision of good accuracy due to the absence of effects from gate-drain and gate-source capacitances, and good stability due to the fixed operating point for transistors. Carrier density and electric field inside the sensor transistor are maintained at a constant value even when there is a change in *V_IN_*.

**Figure 5.**
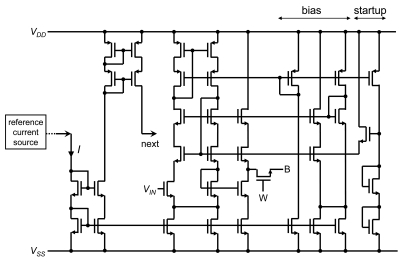
Implemented CMOS source-drain follower.

**Figure 6.**
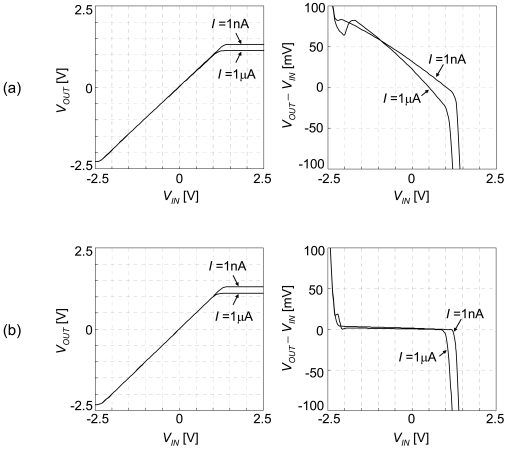
Simulated characteristics of (a) non-cascode and (b) cascode CMOS source-drain followers.

In the integrated sensor array, area is one of the most important factors. When small-sized transistors are used, performance is degraded by the channel modulation effect. A typical method for reducing this effect without significantly increasing the area is cascode topology. We designed wide-swing cascode current mirrors, as shown in Figure 5 [27]. Bias voltage of n-type wide-swing cascode transistors is generated by the output voltage of the voltage follower itself. The number of transistors increases to double, but the increase in sensor cell area is only 50%. The simulation results are shown in Figure 6. Keeping the same operation range, accuracy is improved by more than 100.

In the circuitry of Figure 5, a startup circuit is included to prevent the transistors from entering into the deep cutoff state. Individual current *I* is copied from a reference current source formed at the periphery of the sensor array. The entire current *I_DD_* is 9*I*.

**Figure 7.**
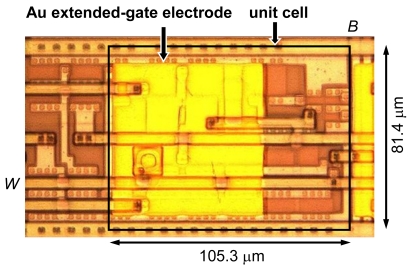
Optical photograph of the fabricated CMOS source-drain follower after formation of the Au extended-gate electrode.

The ISFET sensor array was fabricated using the ON Semiconductor 1.2-μm 2-metal standard CMOS process, as shown in Figure 7. The accuracy *ΔV* = *V_OUT_* − *V_IN_* was mainly determined by the threshold voltage mismatch of the pair of transistors. The variation in threshold voltage is proportional to (*LW*)^−1/2^. In a minimum-sized transistor with *L* = 1.48 μm and *W* = 2.96 μm, the standard deviation *σ_VT_* of the threshold voltage mismatch of the pair of transistors is around 30 mV. In the designed CMOS source-drain follower, the transistor size is increased to *L* = 5.92 μm and *W* = 12.95 μm, to reduce *σ_VT_* to 8 mV. The measured accuracy *ΔV* of five chips is −0.4, −0.5, −3.1, −3.7, and −4.5 mV. Unit cell size is 105.3 × 81.4 μm^2^.

**Figure 8.**
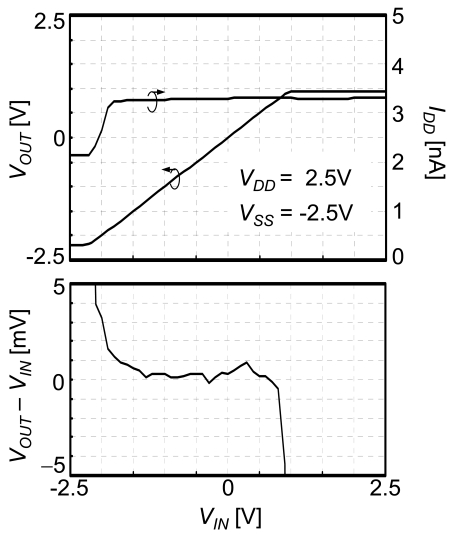
Measured output voltage, total current, and accuracy of CMOS source-drain follower.

**Figure 9.**
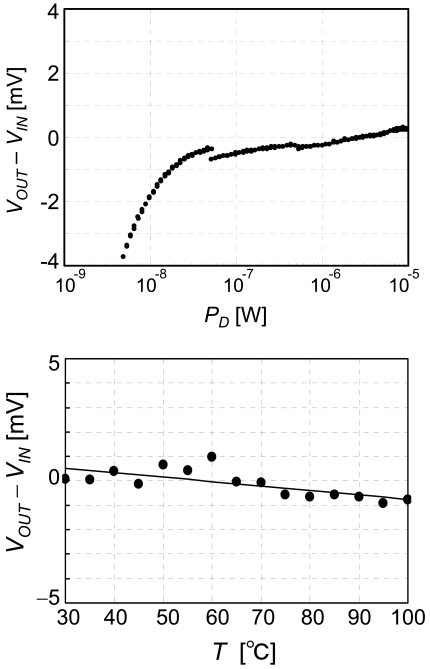
Accuracy measured as functions of power dissipation and temperature. Input voltage *V_IN_* is 0 V. Temperature dependence was taken at *P_D_* = 100 nW.

The measured characteristics of the CMOS source-drain follower are shown in Figure 8. The power supply voltages are *V_DD_* = 2.5 V and *V_SS_* = −2.5 V, the current of the whole circuit *I_DD_* is 3.3 nA (*I* = 370 pA), and the input voltage *V_IN_* changes between −2.5 and 2.5 V. The output voltage and the accuracy of the voltage follower are plotted in the figure. The range used for biosensing is −1.5 to 0.5 V, where the output voltage is equal to the input voltage within 2 mV. As shown in Figure 9, accuracy of 2 mV is confirmed at power dissipation from 10 n to 10 μW and at temperatures between 30 and 100 °C. The temperature coefficient is 2 × 10^−5^ V/°C.

**Figure 10.**
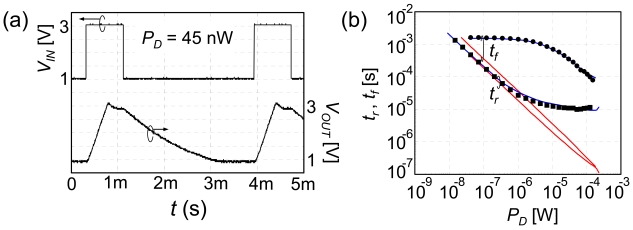
Transient response of CMOS source-drain follower: (a) measured waveform, (b) rise and fall time measured as a function of power dissipation. Blue and red lines correspond to the simulation results in the measured and array configurations, respectively.

Transient step response was measured, as shown in Figure 10. A CMOS source-drain follower was measured through a ceramic package, printed circuit board, and oscilloscope voltage probe. External capacitance and resistance are attached to the output node. In the array configuration, output buffer circuits are formed at the periphery of the array, and the capacitance and resistance on the output node can be substantially reduced. Assuming a bit line capacitance of 400 fF, the response time is estimated as 3 ms at 10 nW and 3 μs at 10 μW.

## 3. ISFET Sensor Array

**Figure 11.**
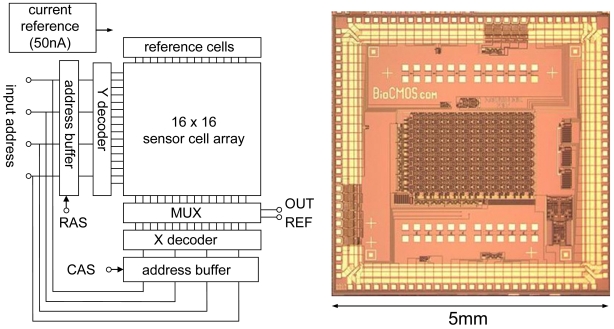
Example of 16 × 16 integrated ISFET sensor array with peripheral circuits.

Using the CMOS source-drain follower, a 16 × 16 ISFET sensor array was designed and fabricated, as shown in Figure 11 [28]. The circuit includes peripheral circuits consisting of a current reference of 50 nA, address buffers, X and Y decoders, and a multiplexer (MUX). The number of input and output ports is 10, power supply *V_DD_* and *V_SS_*, four input addresses, RAS (raw address strobe), CAS (column address strobe), and output voltage of sensor and reference cells. Total power consumption is 0.5 mW.

In the biosensor experiments, the rather large chip size is convenient for facilitating the application of aqueous solution. The 1.2-μm CMOS process is suitable for the experiments, since the chip size is adequate and its cost is low. The 6-inch wafers are fabricated by standard process at a semiconductor factory.

**Figure 12.**
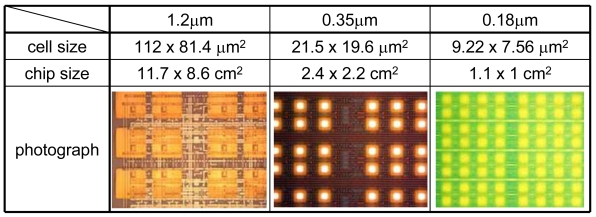
Cell size, chip size for 1,024 × 1,024 array, and optical photograph of fabricated chips using 1.2-, 0.35-, and 0.18-μm standard CMOS processes.

When the array size is increased, the chip area, readout time, and power consumption also increase. The use of a finer fabrication process reduces the chip size. Figure 12 shows the cell size, estimated chip size for a 1,024 × 1,024 array, and an optical photograph of fabricated chips using 1.2-, 0.35-, and 0.18-μm standard CMOS processes.

**Figure 13.**
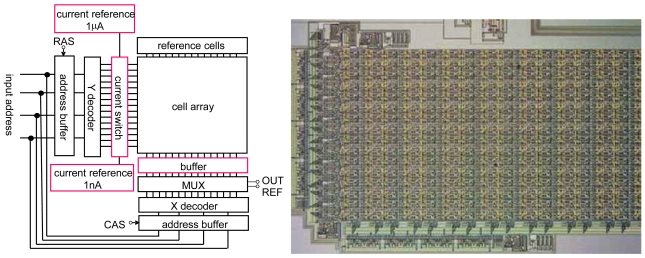
Experimental chip for high-speed operation and low power consumption. Chip with a 32 × 32 array was fabricated by 1.2-μm standard CMOS process.

One of the methods for achieving high-speed, low-power operation is such that high current is supplied to the readout cells for high-speed operation and low current is supplied to the other cells to reduce power consumption. Also, a buffer circuit at each bit line will reduce the output load of the sensor cell, resulting in high-speed readout. Using the example of a 1,024 × 1,024 array, when the power dissipation is 5 μW for selected cells and 5 nW for unselected cells, the total power consumption is around 100 mW, and all data can be obtained within 0.1 s. The experimental chip is shown in Figure 13.

The following experiments employed the 16 × 16 array shown in Figure 11.

## 4. pH Detection

**Figure 14.**
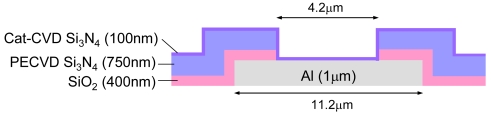
Schematic cross section around the surface of a chip for pH detection.

The pH value is vital information related to human health. Many different biosensors have been developed based on pH sensors since various biomolecular interactions produce protons. Silicon nitride is commonly used as a pH sensing layer. Low-pressure chemical vapor deposition (LPCVD) supplies high-quality silicon nitride, but it cannot be applied after metallization due to the high temperature required. Plasma-enhanced CVD (PECVD) is a low-temperature process, but the deposited silicon nitride is not dense and has poor chemical tolerance to aqueous solutions. Catalytic CVD (Cat-CVD) is a low-temperature process and the deposited silicon nitride is of high quality similar to that obtained by LPCVD [29,30]. In the Cat-CVD method, deposition gases are decomposed by catalytic cracking reaction with a heated catalyzer placed near the substrate. The films are deposited without any help from plasma or photochemical excitation [31]. In the deposition of silicon nitride, a silane (SiH_4_) and ammonia (NH_3_) mixture is used as the deposition gas, with a back pressure of 8.5 × 10^−7^ Torr, silane flow rate of 6 sccm, ammonia flow rate of 300 sccm, catalyzer temperature of 1,700 °C, and substrate temperature of 350 °C. In the following experiment, a 100-nm silicon nitride layer was deposited [32]. A schematic cross section around the chip surface is shown in Figure 14.

**Figure 15.**
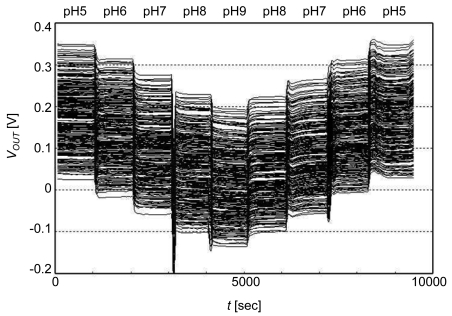
pH detection by a 16 × 16 ISFET sensor array. The voltage of the reference electrode is 0 V.

Following silicon nitride deposition, the chip was mounted on a ceramic quad flat package (QFP) and bonded by wire. An acryl bath was placed on the chip with an Ag/AgCl reference electrode soaked in KCl saturated solution enclosed in glass [see Figure 19(a)]. Mixing 0.1 M K_2_HPO_4_ (pH 9.0) and 0.1 M KH_2_PO_4_ (pH 4.4), solutions with pH 5, 6, 7, 8, and 9 were prepared and 4.4 ml of solution of each pH was injected into the bath. The pH value was changed successively from pH 5 to pH 9 and then reversed from pH 9 to pH 5. Measurement time for each pH value was about 1,000 s. There was no solution in the bath between the injecting and draining solution. The results are shown in Figure 15 [32]. The output voltage from each sensor cell is scattered at ∼0.3 V since the electrodes are electrically floating and a different amount of charge accumulates on each electrode during fabrication [9,17]. UV exposure could reduce the variation in charge. Slow pH response was observed, which may be attributed to buried sites in the hydrated surface layer and anodic surface oxidation [33].

**Figure 16.**
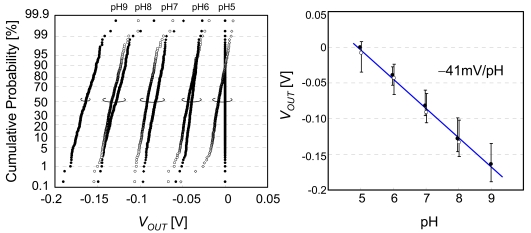
Cumulative probability of output voltage, and median ±3σ plot as a function of pH. From pH 5 to 9 (black) and pH 8 to 5 (white). The output voltage is the result after subtracting the initial values in order to eliminate the charge effect from the floating gate.

**Figure 17.**
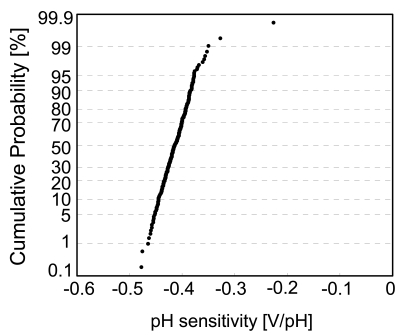
Cumulative probability of pH sensitivity of individual sensor cells.

The cumulative probability of output voltage, after subtracting the initial values, is plotted in Figure 16, as projected by the inverse cumulative standard normal distribution function. The plot becomes a straight line if the distribution is Gaussian. Figure 16 shows that the distribution is well described by a Gaussian distribution with 1% abnormality. The standard deviation is 4–9 mV, which could be reduced by increasing the sensing area *S* from 4.2 × 4.2 μm^2^, since the standard deviation is proportional to *S*^−1/2^. The cumulative probability of pH sensitivity of individual sensor cells is plotted in Figure 17. The median of pH sensitivity is −41 mV/pH, which is smaller than the theoretical value of −57 mV/pH. The reason for the lower sensitivity may be explained by the oxygen-rich layer on the Si_3_N_4_ surface. The major sites on the Si_3_N_4_ surface are silanol (SiOH) and amine (SiNH_2_) sites [34]. From the site-binding model and Nernstian equation, pH sensitivity is given by a function of the site density of silanol and amine [33,35]. The presence of an oxygen-rich surface layer increases the silanol sites and degrades the pH sensitivity [36]. It is reported that treatment in hydrofluoric acid (HF) provides the silicon nitride surface with high pH sensitivity, but this treatment is only effective if carried out immediately prior to testing [34]. The pH response of samples etched by HF and tested after 24 h was found to be similar to that of a sample that was not etched by HF due to anodic surface oxidation. Several surface treatments to improve the pH sensitivity have been investigated, such as O_2_ plasma treatment [37].

## 5. DNA Detection

**Figure 18.**
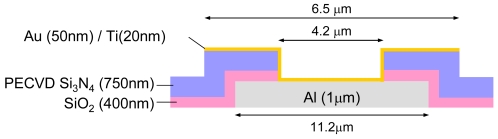
Schematic cross section around the surface of a chip for DNA detection.

Using the integrated ISFET sensor array, preliminary experiments on DNA detection were performed. Gold extended-gate electrodes were used to immobilize the probe DNA. After fabrication of the chip using the standard CMOS process, the chip surface was cleaned with acetone, IPA, and deionized (DI) water to remove dirt such as organic matter and oils, followed by the deposition of 20-nm-thick Ti and 50-nm-thick Au layers. Then, extended-gate electrodes were patterned by optical lithography and wet etching using AURUM-301 (Kanto Chemical) and WLC-T (Mitsubishi Gas). A schematic cross section is shown in Figure 18.

**Figure 19.**
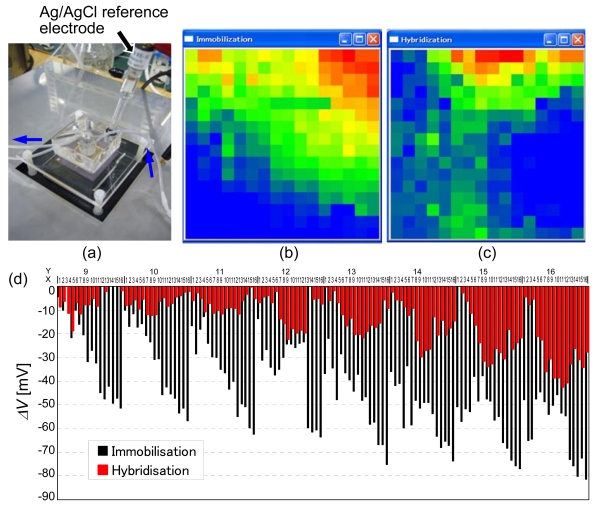
Preliminary experiment on DNA detection using a 16 × 16 ISFET sensor array; (a) setup of experiment, distribution of output voltage change by (b) immobilization, (c) hybridization, (d) output voltage change before/after immobilization (black) and before/after hybridization (red).

Immobilization of a 5′-thiol-modified oligonucleotide of 20-mer GGGAAAAAAAAAAAAAAGGG, and hybridization with the complementary oligonucleotide CCCTTTTTTTTTTTTTTCCC were detected in a 1-mM phosphate buffer (pH 7.0) containing 1 mM NaCl and 1 μM ethylene diamine tetraacetic acid (EDTA), as shown in Figure 19 [28]. The adsorbed quantity of probe DNA is estimated to be 1.3 × 10^−11^ mol/cm^2^ by quartz crystal microbalance (QCM) measurement using a gold resonator. Biomolecular interactions were observed as the time development of two-dimensional distribution. Maximum voltage change was 80 mV for immobilization and 40 mV for hybridization. In this experiment, the uniformity of biomolecular interactions was not good. One reason is the non-uniform fluid flow. The uniformity could be improved by forming microfluidic channels on a chip [38]. Another reason is the exchange of electrons between the extended-gate electrode and electrolyte. Long-term drift of sensed voltage was observed as 100 mV/h. On the other hand, the drift was reduced to 2 mV/h when Cat-CVD silicon nitride was deposited on the extended-gate electrode.

DNA detection was performed using the silane-coupling method for probe immobilization on silicon nitride using the same formula described in [19]. The results show voltage changes of around 100 mV for probe immobilization, 12 mV for hybridization of complementary target DNA, and less than 1 mV for reverse-complementary target DNA. These results are similar to the values of 78 mV for immobilization and 11 mV for hybridization reported in [19]. The immobilization density on silicon nitride is one or two orders smaller than that on gold electrode. The output voltage difference, however, depends on the shape of the oligonucleotides, and higher density does not mean higher output voltage. Also, it should be noted that the dependence of capacitance between DNA and extended-gate electrode is weak, similar to the situation where the threshold voltage shift of a FLASH memory transistor is almost independent of the gate insulator thickness.

## 6. PSI Photosensor

Photosensors are widely used in both everyday-life products and advanced technologies. Although inorganic materials are used in the photosensors of conventional electronic devices, their performance is not satisfactory with regard to energy efficiency such as quantum yield [39].

The quantum yield of the photoelectric conversion of photosynthesis is 100% [24], which is extremely difficult to achieve by artificial means. In addition, thermal energy is extremely suppressed, and the biological molecules are low in cost with a low environmental load so that material production and postprocessing are easy. By incorporating living body materials and an integrated circuit, a completely new type of high-density photosensor with high sensitivity can be realized, which is not possible using semiconductor technology alone. A single photon can be detected at room temperature using such a system combined with a field effect transistor (FET) with a nanogate.

This section describes photo detection using an ISFET sensor array and a photosynthesis protein complex, Photosystem I (PSI) (a photoreceptor macromolecule).

**Figure 20.**
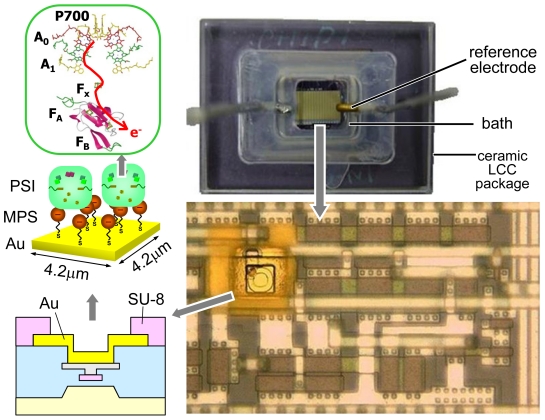
Chip assembly and optical photograph of PSI photosensor after SU-8 formation.

The PSI in chloroplast is extracted from *Thermosynechococcus elongatus*, the diameter of which is approximately 10 nm. The PSI includes reaction center chlorophyll a (P700), chlorophyll a (A0), phylloquinone (A1), and Fe-S clusters F_X_, F_A_ and F_B_, which transmit electrons. Isolation and refinement of PSI were performed as previously reported [40]. Figure 20 shows PSI modification on the extended-gate electrode of a transistor. A self-assembled monolayer (SAM) of 3-mercapto-1-propanesulfonic acid sodium salt (MPS) covers the Au electrode. The SAM has a negative electric charge, and the ferredoxin around the Fe-S clusters of the PSI has a positive electric charge. As a result, the PSI is electrostatically fixed on the electrode. According to preliminary investigations involving QCM measurements using gold resonators, the adsorbed quantity was estimated to be 4.34×10^−10^ mol/cm^2^ for MPS, and 7.7 × 10^−13^ mol/cm^2^ for PSI.

In measuring the photoresponse, sodium L-ascorbate (NaAs) and 2,6-dichloroindophenol (DCIP) are used as electrolyte for transporting electrons. DCIP becomes a reducer carrying electrons to P700 in PSI when it is reduced by NaAs. Under light irradiation, electrons on P700 are excited to higher energy levels (energy gap is 1.77 eV corresponding to wavelength of 700 nm). The excited electrons are transferred along the electron transport chain and finally trapped at the electrode by tunneling through the SAM film. In this manner, PSI acts as a pump to supply electrons to the electrode, and the electric potential change in the gate electrode is detected by the ISFET sensor.

The silicon nitride formed by standard CMOS process as the top layer (see Figure 18) can easily trap electronic charges in the solution, causing electric potential drift around 1 mV by light. To reduce the light-induced drift, a protective film that does not trap electrons should be used as a covering. For the protective film, SU-8 (an epoxy-based negative resist) was adopted. First, an SU-8 layer of 9 μm was spin-coated. Then, the SU-8 was patterned by UV exposure. The active area of the electrode pad was very small because the thin film was left on the Au electrode surface after development. Therefore, O_2_ plasma etching for 5 minutes on a chip was undertaken to remove the residue of SU-8. As a result, the active area of the electrode measured by cyclic voltammetry (CV) was about 5.3 μm^2^, around 30% of the electrode area. The light-induced drift was reduced to less than 0.1 mV.

The photoresponse of the CMOS source-drain follower itself was measured using light irradiation produced by RGB light emitting diodes (red: 2.4 mW/cm^2^, green: 1.11 mW/cm^2^, blue: 3.28 mW/cm^2^) inside a shielding box. Light irradiation induces photocurrent in the circuit. When the current *I* is small, the effect is particularly marked, so that the photoresponse, defined by *ΔV_OUT_* = −*V_OUT_* (light ON) + *V_OUT_* (light OFF), becomes large. For a condition in which the effect of the photocurrent in the circuit could be ignored, current *I* of 0.5 μA was chosen in the following PSI photosensor measurement, where photoresponse *ΔV_OUT_* is less than 0.4 mV [23].

After the formation of electrodes on the CMOS chip, it is mounted on a ceramic leadless chip carrier (LCC) package and bonded by wire. It is necessary to dip only the chip surface into solution for modification and evaluation of the PSI photosensor. Silicone is employed to protect the bonding wire. Then, the PSI photosensor is formed as follows. The chip is dipped in 10 mM MPS solution for 2 h. After washing in DI water and 2-morpholinoethanesulfonic acid (MES) buffer [20 mM MES-NaOH (pH 6.4)/0.02% (w/v) n-dodecyl-β-d-maltoside (β-DM)], the electrode is dipped in 2.8 mg/mL PSI solution and maintained at 4°C for four days. It is washed in MES buffer without drying the electrodes. A bath with a reference electrode of platinum painted with Ag/AgCl paste is placed on the chip as shown in Figure 20. Next, 20 mM MES-NaOH buffer (pH = 6.4), 100 mM NaClO_4_, 250 mM NaAs, and 25 mM DCIP are placed in the bath.

The chip is set on an XYZθ stage in a shielding box. To drive the circuits, DC voltage is supplied to the chip by a semiconductor parameter analyzer. A halogen lamp is used as a light source, and the wavelength is selected by a monochromator. An optical fiber guides the light into the shielding box, and light having a diameter of 70 μm is focused on the chip from the upper part of the XYZθ stage. The electrical signal caused by irradiation is read by a multimeter. *V_REF_* = 0 V is applied to the reference electrode, and the output voltage of the CMOS source-drain follower is measured.

**Figure 21.**
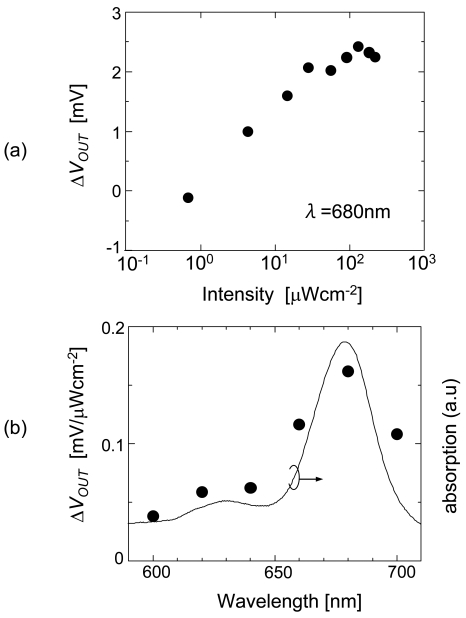
(a) Intensity dependence of photoresponse and (b) action spectrum of PSI photosensor, compared with the absorption spectrum of PSI.

While the electrons from the PSI are trapped in the electrode, O_2_ in the solution near the electrode extracts the electrons. The electric potential is determined by the balance between the input and output of electric charge. Figure 21 (a) shows the photoresponse *ΔV_OUT_* as a function of light intensity. The photoresponse of PSI saturates at 30 μW/cm^2^. Increasing the intensity increases the number of electrons activated by PSI, which changes the output voltage in the negative direction. The limit for transferring electrons to P700 from DCIP [41] is reached when the intensity is increased and the output voltage is saturated.

Figure 21 (b) shows the PSI action spectrum of the PSI photosensor. The wavelength was changed at 20-nm intervals from 600 to 700 nm while turning the light on and off every 90 s. The change in output voltage per 1 μW/cm^2^ change in intensity is shown. At a wavelength of 680 nm, there is a change of approximately 0.18 mV per 1 μW/cm^2^. The wavelength dependence fits well with the absorption spectrum of PSI.

**Figure 22.**
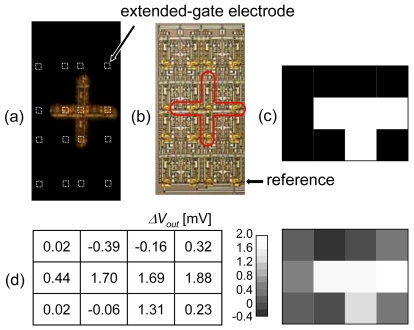
Imaging with the sensor array. (a) Optical photograph of illuminated pattern. (b) Microphotograph of 4 × 4 sensor array. In each column, the bottom cell is used as a reference for the other three cells. (c) Expected output pattern. (d) Measured photoresponse.

To apply the photosensor as an image sensor, a 4 × 4 sensor array was measured [42]. The light without a monochromator was patterned by a metal slit, as shown in Figure 22 (a). To reduce the drift effect, the output voltage of each cell was subtracted by the value of the corresponding reference cell. Figure 22 (d) shows the obtained image. The photoresponse of four illuminated cells ranged from 1.3 to 1.9 mV, and the change in other cells not illuminated was −0.4 to 0.4 mV.

## 7. Conclusions

An integrated ISFET sensor array was designed using CMOS source-drain followers, and fabricated by the standard 1.2-μm CMOS process. Accuracy of 2 mV except for the threshold voltage mismatch was obtained at power dissipation between 10 n and 10 μW and at temperatures between 30 and 100 °C. The area of the sensor cell is 105.3 × 81.4 μm^2^, which can be reduced by using fine process technology. In a 0.18-μm process, the cell size is reduced to 9.22 × 7.56 μm^2^, and a 10^6^ sensor array can be constructed on an 11 × 10 mm^2^ chip with power consumption of 100 mW.

For pH detection, a Cat-CVD silicon nitride layer was formed on the electrodes, and −41 mV/pH sensitivity was obtained. Standard deviation was 4–9 mV for a 4.2 × 4.2-μm^2^ sensing area, which might be attributed to non-uniformity of silicon nitride thickness due to the rough electrode surface.

For DNA detection, thiol-modified oligonucleotides were immobilized on Au extended-gate electrodes. The immobilization and hybridization processes were observed as the time development of two-dimensional distribution. Long-term drift of extended-gate electrode voltage was very large. Silane-coupling method on silicon nitride and microfluidic channels is needed to suppress the drift and improve the detection accuracy.

A new photosensor was demonstrated by integrating PSI on a CMOS chip. A maximum signal of 3 mV, namely, the change in the output voltage caused by light irradiation on the PSI photosensor, was detected. This signal is 12% of that obtained using a large electrode of 16 mm^2^; the reduction may have been caused by the coverage of PSI, especially near the edge of the electrode. Light selectivity was confirmed and no interference was observed between the cells in a sensor array. In this experiment, native PSI was used. High sensitivity and fast response are expected using PSI reconstructed by molecular wire [25,40]. Since the quantum yield of the photoelectric conversion of photosynthesis is 100%, a single photon could be detected using the proposed system combined with a nanogate FET or a single-electron transistor.
